# EzMAP: Easy Microbiome Analysis Platform

**DOI:** 10.1186/s12859-021-04106-7

**Published:** 2021-04-07

**Authors:** Gnanendra Shanmugam, Song Hee Lee, Junhyun Jeon

**Affiliations:** 1 Department of Biotechnology, Yeungnam University, Gyeongsan, 38541 Gyeongbuk Korea; 2 Plant Immunity Research Center, Seoul National University, Seoul, 08826 Korea

**Keywords:** Microbiome analysis platform, Microbiome data analysis, QIIME2 analysis, Microbiome user-friendly tool, Microbiome data visualization

## Abstract

**Background:**

The rapid advances in next-generation sequencing technologies have revolutionized the microbiome research by greatly increasing our ability to understand diversity of microbes in a given sample. Over the past decade, several computational pipelines have been developed to efficiently process and annotate these microbiome data. However, most of these pipelines require an implementation of additional tools for downstream analyses as well as advanced programming skills.

**Results:**

Here we introduce a user-friendly microbiome analysis platform, EzMAP (Easy Microbiome Analysis Platform), which was developed using Java Swings, Java Script and R programming language. EzMAP is a standalone package providing graphical user interface, enabling easy access to all the functionalities of QIIME2 (Quantitative Insights Into Microbial Ecology) as well as streamlined downstream analyses using QIIME2 output as input. This platform is designed to give users the detailed reports and the intermediate output files that are generated progressively. The users are allowed to download the features/OTU table (.biom;.tsv;.xls), representative sequences (.fasta) and phylogenetic tree (.nwk), taxonomy assignment file (optional). For downstream analyses, users are allowed to perform relative abundances (at all taxonomical levels), community comparison (alpha and beta diversity, core microbiome), differential abundances (DESeq2 and linear discriminant analysis) and functional prediction (PICRust, Tax4Fun and FunGuilds). Our case study using a published rice microbiome dataset demonstrates intuitive user interface and great accessibility of the EzMAP.

**Conclusions:**

This EzMAP allows users to consolidate the microbiome analysis processes from raw sequence processing to downstream analyses specific for individual projects. We believe that this will be an invaluable tool for the beginners in their microbiome data analysis. This platform is freely available at https://github.com/gnanibioinfo/EzMAP and will be continually updated for adoption of changes in methods and approaches.

## Background

Microbiome analyses based on targeted amplicon sequencing provide valuable insights into diversity and functions of microbial communities [1]. The rapid advances in sequencing technology have enabled the researchers to explore the complex microbial communities at an unprecedented resolution [2]. The amplicon sequences are used to identify taxonomic groups in the samples [3]. The resulting taxonomic data are used to elucidate their relative abundances, and to calculate diversity measures of communities such as alpha- and beta-diversity. Such studies need a series of computational processes such as sequence quality filtering, sequence alignments, and phylogeny building, which can be accomplished by some dedicated databases and common bioinformatics tools. [4]. However, the other processes such as quantification of community-profile similarity and taxonomic classifications requires specialized databases such as SILVA [5], Ribosomal Database Project (RDP) [6], EzBioCloud [7] and Greengenes [8], and tools like QIIME [9] and Mothur [10] that are designed specifically for marker-gene analyses. The analysis based on target genes can provide important insights on community functions that cannot be obtained through analyses based on barcoding genes. Such analyses of community functions require specialized tools and database as well. Some parts of marker gene analysis pipelines can be used for metagenomic studies based on the well-conserved key genes in ecologically important pathways that are involved in carbon and nitrogen cycling.

The core step in microbiome analysis is the taxonomic classification of the representative sequences and clustering into OTUs (Operational Taxonomic Units). The most popular pipeline for amplicon sequencing is QIIME2 and Mothur. Although in popular use, these pipelines require implementation of additional tools for their downstream analyses as well as basic programming skills, which may discourage use by researchers with little bioinformatics expertise. Making QIIME2 user-friendly and accessible to researchers, therefore, requires graphical user interface (GUI) that allows novices to upload fastq files, choose denoising algorithms and reference databases to perform OTU clustering in a few clicks.

In this paper, we introduce a user-friendly microbiome analysis platform, EzMAP that was developed using Java Swings, Java Script, and R programming. This tool provides GUI allowing use of QIIME2 functionalities for metadata profiling, read pre-processing, sequence processing and classification, OTU (operational taxonomic unit) clustering, taxonomy assignment, and visualization. QIIME2 output files can be channelled to downstream analyses within the EzMAP framework.

### Implementation of EzMAP

**Fig. 1:**
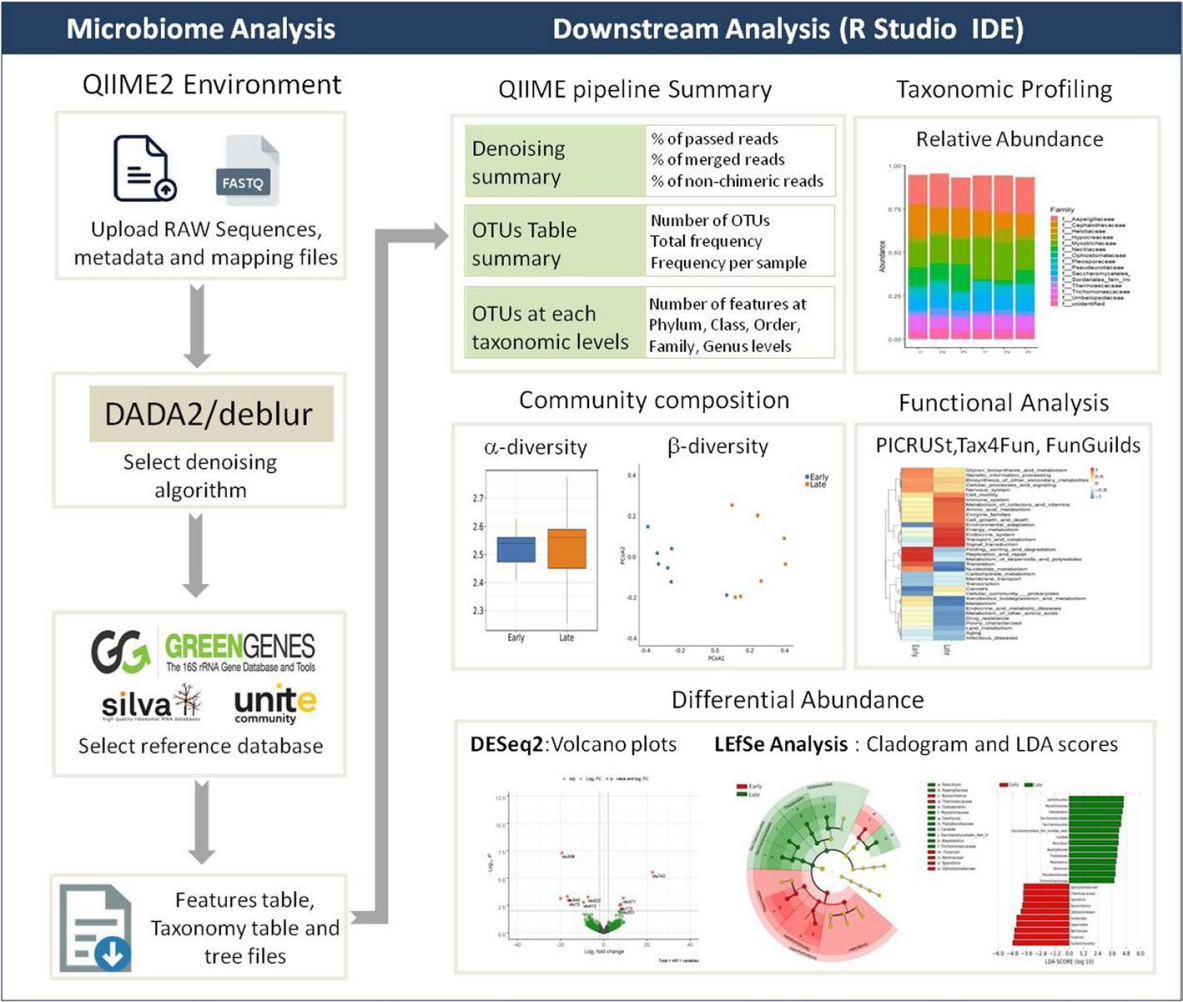
A diagram showing EzMAP workflow

EzMAP provides comprehensive and streamlined workflow for metagenome projects using 16S rRNA and ITS1/ITS2 sequence data, ranging from pre-processing of raw sequence data to downstream analyses and visualization. The design of this platform (Fig. 1), help the users to overcome the burden of command-line usage, which is prone to errors resulting from typos and parameter settings. In EzMAP, the users are allowed to locate the working directory and to upload the manifest file (path to fastq files), meta-data files. Upon file uploads, input files are automatically validated for proper file format. In order to obtain high-quality representative sequences while filtering the poor quality sequences, the users are provided with the choice of DADA2 [11] and Deblur [12] algorithms through QIIME2. As a next step, the non-chimeric sequences are searched against a known reference taxonomy classifier with a threshold of 97% similarity and 70% confidence level expressed as 0.7 in QIIME2 as default settings for OTU clustering [13]. The users are provided with the selection of publicly available databases such as SILVA, Greengenes, and UNITE [14]. The users are also provided with the option to train their custom classifiers using q2-feature-classifier protocol from QIIME2.

The MAFFT program [15] is used to perform multiple sequence alignment and construction of phylogenetic tree. The users are finally provided with features/OTU table (.biom;.tsv;.xls), representative sequences (.fasta), phylogenetic tree (.nwk) and taxonomy assignment file (optional) to download or to perform further downstream analyses along with provenance logs for each step in the pipeline. The final output is the OTU table, which is a matrix of sequence counts or taxa per-sample and typically a primary input for downstream analyses. EzMAP provides various options for downstream analyses such as relative abundance at all taxa levels, alpha- and beta-diversity measures along with visualizations. The differential abundances for individual taxa can also be performed through the wrapper scripts for DESeq2 [16] and Linear discriminant analysis Effect size (LEfSe) [17]. Furthermore, EzMAP is provided with functional analysis options through the wrapper scripts for PICRust [18], Tax4fun [19] and FUNGuild [20]. For such tasks to be performed, OTU tables are transformed into suitable data structures for further analysis and visualization using RStudio IDE (integrated development environment) embedded in EzMAP.

EzMAP combines all necessary packages and tools to perform microbiome analysis, and thus helps users to avoid complicated and time-consuming installations. We have produced a straight-forward microbiome analysis platform that utilizes QIIME2 tools to perform the major steps of 16S and ITS1/2 amplicon analysis. We have written wrapper scripts using JavaScript and bash to upload the fastq files and to set the options like selection of denoising algorithm, the reference database for OTU clustering and to seamlessly integrate multiple downstream analyses tools. EzMAP is primarily designed to be used interactively through the Linux and Mac terminals, while on windows it is executed through docker containers. The EzMAP docker container includes all the packages required for the execution of pipeline and allows users to run their analyses by installing docker along with JAVA. EzMAP is freely accessible to the global microbiome research community at https://github.com/gnanibioinfo/EzMAP.

## Results and discussion

### Overview of EzMAP

EzMAP is designed to serve as a comprehensive data analysis platform to perform both upstream and downstream analyses of 16S rRNA and ITS marker gene datasets. This platform is intended to minimize or eliminate the use of command-line arguments during the data processing. EzMAP simplifies the upstream and downstream processing with user-friendly GUI, which can be efficiently executed by novice microbiome researchers. In the current release of EzMAP, the classification of the 16S rRNA gene and ITS sequences in the upstream module is facilitated by QIIME2. EzMAP does not require installation of any docker containers to run on Mac and Linux operating systems, while it requires installation of docker containers on Windows OS to execute the upstream analysis (pre-processing of sequences and binning). EzMAP is designed to pull the QIIME2 docker images on a Windows OS, which requires more disk space. The ability of EzMAP to deploy high-end clusters or Windows OS running on high-end computers with more cores makes it easier to run all analyses. The instruction for docker installation on Windows OS for EzMAP adoption is made available in README files. The EzMAP has flexibility to execute the downstream analyses as a separate module on any OS platforms including Windows OS without installing docker container.

EzMAP supports the pre-processing of marker gene-based analyses. In the upstream analysis, the Illumina fastq reads are taken in as input files, and OTU table and taxonomy table are produced as output. The pipeline implemented in EzMAP is mainly based on QIIME2, the most widely used microbiome analysis pipeline. At every step, the users are allowed to change the default parameters and can select their own choice of settings. Double-click of EzMAP icon automatically activates EzMAP environment to download the updated versions of SILVA database as default reference databases for taxonomic classification.

EzMAP uses DADA2 for a quality control of sequences, and uses updated SILVA database and classifier for clustering, classification and taxonomy assignments of representative sequences into OTUs as a default parameters. The biom file consisting of OTU table and taxonomy table and metadata file and phylogenetic tree as tree file (.nwk) are the final outputs of upstream analysis.

The resulting biom file from upstream module is fed as an input file into downstream module, which automatically converts the biome file into phyloseq object for further analysis and visualization using RStudio IDE. A summary of biom file including the number of taxa, the number of sample variables, and the number of OTUs at each taxonomic level is displayed upon uploading the biom file. EzMAP users are provided with the filter parameters in the downstream analysis to subset and retain the unassigned and unknown sequences. These filter parameters can be applied on various levels of taxonomic classifications and metadata variables to subset the non-bacterial lineages such as chloroplast, mitochondria and archaea. The contents of biome file (OTU table and taxonomy table) along with metadata file can be inspected on the computer screen by users. EzMAP also shows the summary of the total count of OTU abundances and distribution of OTU abundances per community at every taxonomic rank. The rarefaction curves can be visualized as a parts of EzMAP’s primary downstream analysis output.

For alpha diversity analysis, the EzMAP users are provided with the options in estimating the diversity measure of richness and evenness such as Observed, Chao1, ACE, Shannon, Simpson, InvSimpson and Fisher through phyloseq v 1.16.0 [21]. Differences between the alpha diversity of samples are statistically evaluated using Kruskal–Wallis test as default. The beta diversity is computed by ordination distance to compare the similarity/ dissimilarity between the samples. The current version of EzMAP is provided with PCoA methods to calculate the bray–curtis distance, jaccard distance, weighted and unweighted UniFrac methods with the PERMANOVA p-values computed through a R package vegan v. 2.5–6 [22]. EzMAP utilizes the DESeq2 v1.28.1 and microbiomeMarker v. 0.0.1.9 [23] for LEfSe analysis to identify differentially abundant features in the datasets. The results of DESeq2 are plotted as fold-change versus p-values of each OTU by using Enhanced Volcanoplot v. 1.6.0 [24]

EzMAP also provides the functional prediction of OTU through Tax4Fun (for OTU table generated by using SILVA database) by using Tax4Fun v 1.0.4, PICRust (for OTU table generated by using Greengenes database) and FunGuild (for 18S rRNA) by using the bash wrappers scripts. All the plots are generated and visualized by using ggplot2 v. 3.3.2 [25]. EzMAP is provided with the flexibility to choose between the upstream data processing and wide range of downstream analyses with visualizations in a single embedded R Shiny App.

By providing easy interface and great flexibility, the EzMAP platform would serve as an invaluable tool for the beginners in microbiome data analysis. Several web-based or desktop applications have been developed over the last decade to support the analysis of microbiome data. Most of these tools have been developed primarily using Mothur and QIIME2 pipelines [26]. As QIIME 2 has established as a de facto standard microbiome analysis workflow/pipeline, the EzMAP wrapped with QIIME2 workflow for upstream analysis would be a standardized and reproducible platform for microbiome data analysis. Additionally, we benchmarked our EzMAP functionalities with other pipelines developed for the same purpose. The runtime for upstream analysis via QIIME2 workflow is consistent across all the platforms. The comparison of EzMAP functionalities in-terms of installation and easy usage with other pipelines developed for the same purpose are summarized in Table 1.

**Table 1:** Summary of comparison of EzMAP with some of the pre-existing microbiome analysis tools (standalone programs only)

Features	EzMAP	QIIME studio	GenePiper	BiomMiner	iMAP
Installation					
Command-line interface	Partial	Yes	No	Yes	Yes
Graphical user interface					
Upstream analysis	Yes	Yes	Not applicable	No	No
Downstream analysis	Yes	Yes	Yes	Yes	Yes
Upstream analysis					
Sequence pre-processing	Yes	Yes	No	Yes	Yes
OUT cluster and taxonomy assignments	Yes	Yes	No	Yes	Yes
Data Summary	Yes	Yes	No	Yes	Yes
Analysis run time (Approx. Hrs)	8:24	8:20	–	7:50	8:18
Downstream analysis					
Alpha diversity	Yes	Yes	Yes	Yes	Yes
Beta diversity: Ordination plots	Yes	Yes	Yes	Yes	Yes
Basic statistics	Yes	Yes	Yes	Yes	Yes
Differential abundance analysis					
LefSe	Yes	No	No	No	No
DESeq2	Yes	No	No	Yes	No
Functional analysis					
PICRUSt	Yes	No	No	No	No
Tax4Fun	Yes	No	No	No	No
FunGuild	Yes	No	No	No	No

BiomMiner [27] and iMAP [28] are primarily embedded with Mothur and QIIME 2 for sequence processing and classification. The downstream analysis and visualization of these platforms is implemented via R language. GenePiper [29] focuses only on downstream analysis and data visualization. Unlike these platforms, EzMAP provides streamlined analysis flow seamlessly combining upstream analysis through QIIME2 and downstream analyses with additional features such as differential abundance using DESeq2, LEfSe, functional prediction using Tax4Fun, PICRust and FunGuild, and visualization using RStudio IDE. We are planning to update EzMAP annually with additional functionalities such as network analysis and machine learning pertaining to microbiome analysis and to add more options at every step in downstream analyses to produce interactive charts. EzMAP is provided with installation instructions, example datasets, and sample plot images to facilitate quick evaluation and adoption of the platform at https://github.com/gnanibioinfo/EzMAP.

### Reproducible case study

For demonstration of EzMAP usage, we used the published dataset of Edwards’s et al*.,* 2015 [30]. This dataset was used to study the structure and variation of root-associated microbiome of six cultivated rice varieties collected from three different rice fields across the Central Valley of California. For EzMAP demonstration, we used a subset of 36 samples belonging to two compartments (Rhizosphere and Root Endosphere) of two indica varieties IR50 and 93-11 from their greenhouse experiment.

At first, the raw 16S rRNA sequencing (Illumina MiSeq) reads from 36 samples, were downloaded from the National Center for Biotechnology Information Short Read Archive (accession no. SRP044745). Metadata file describing the samples and variables such as compartment (Rhizosphere and Endosphere), soil_location (Arbuckle, Davis, Sacramento) and rice_cultivar (IR50, 93-11) was manually prepared. The mapping files, which link samples and forward reads to individual experimental variables, were prepared manually as well. EzMAP implemented qiime2-2020.8 functions to process and classify the representative sequences for this case study. The pipeline uses DADA2 as default for denoising algorithm and reports the merged and non-chimeric reads. Taxonomic classification of the representative sequence for each OTU was done using QIIME’s version of the Ribosomal Database Project’s classifier against the Greengenes 16S rRNA database (13_5 release). All non-chimeric sequences were clustered into operational taxonomic units (OTUs) based on 97% pairwise identity using the Greengenes 16S rRNA database as a reference.

**Fig. 2:**
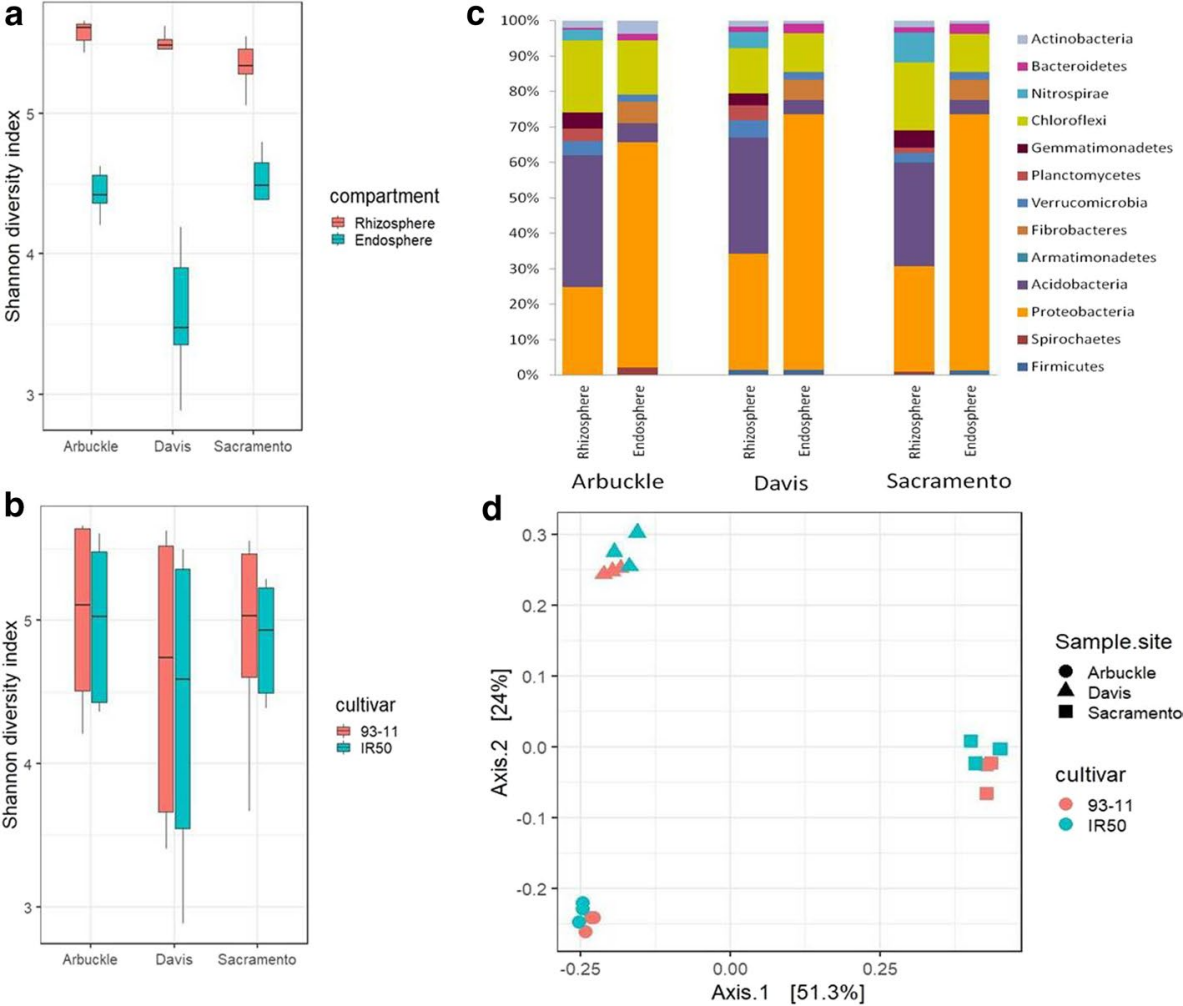
Demonstration of EzMAP usage for microbiome data from Edward et al. **a** Rhizospheric compartments showing higher microbial diversity than endosphere in all the three field samples. **b** Cultivar 93-11 showing alpha diversity comparable to cultivar IR50 in all the three field samples. The horizontal bars within boxes represent median values. **c** Barplot of phylum-level relative abundances in rhizosphere and endosphere from three field samples. **d** PCoA using the weighted UniFrac metric indicates the separation between microbial communities among the three field samples. All the plots are drawn using ggplot2 v3.3.3 in RStudio v.1.3 incorporated in EzMAP

A total of 3,939,881 high-quality reads were obtained with a median read count per sample of 98,272. The high-quality reads were clustered using > 97% sequence identity into 4,280 bacterial OTUs through up-stream analysis. In downstream analysis, discarding the low-abundance OTUs (< 5 total counts) resulted in 838 OTUs. Measures of alpha-diversity showing higher diversity in rhizosphere compared to endosphere (*P* < 0.001) (Fig. 2a) of each soil type. The endosphere microbial communities of Arbuckle and Sacramento field showed higher diversity than the Davis field (Fig. 2b). These results were in accordance with the published data. Higher relative abundance of *Proteobacteria, Acidobacteria* and lower abundances of *Planctomycetes, Spirochaetes* and *Gemmatimonadetes* observed in the endosphere when compared to the rhizosphere compartment (Fig. 2c) were also reproduced by the EzMAP. Similarly, the WUF PCoA (Principal coordinate analysis (PCoA) based on weighted UniFrac metric (WUF)) showed that microbial communities of three different field soils separate across the first principal coordinate (Fig. 2d) (*P* < 0.001, PERMANOVA). Similar structures of microbial communities between rice genotype 93-11 and IR50 when using WUF was also in accordance with the published data. Taken together, all the aforementioned results were consistent with the results reported in the original paper, attesting the utility of EzMAP. Considering the challenges in robustness and reproducibility of microbiome data analysis, the EzMAP would not only improve the reproducibility of microbiome researches but also help the novices to engage in the microbiome data analysis.

## Conclusions

Here we present EzMAP, a user-friendly platform for microbiome analysis. This platform allows users to consolidate the microbiome analysis processes ranging from raw sequence processing to downstream analyses specific for individual projects. We believe that this will serve as a starting platform for the beginners and as all-inclusive package for the advanced users in their microbiome data analysis. This platform is freely available and will be continually updated to adopt new developments in methods and approaches.

## Availability and requirements

- **Project name**: EzMAP (Easy Microbiome Analysis Platform)
- **Project home page**: https://github.com/gnanibioinfo/EzMAP
- **Operating system(s)**: Platform independent
- **Programming language**: Java Swings, Bash and R 4.0
- **Other requirements**: Java JRE 1.8, RStudio v1.3
- **License: **GPL v2.0
- **Any restrictions to use by non-academics**: none

## Data Availability

The project files and test data of EzMAP are available at https://github.com/gnanibioinfo/EzMAP. Project name: EzMAP (Easy Microbiome Analysis Platform). Project home page: https://github.com/gnanibioinfo/EzMAP. Requirements: Java Runtime Environment (JRE). Programming languages: JAVA and R. License: GNU GPL.

## Abbreviations

DADA2: Divisive amplicon denoising algorithm
DESeq: Differential expression analysis for sequence count data
EzMAP: Easy Microbiome Analysis Platform
GUI: Graphical user interface
ITS: Internal transcribed spacer
LEfSe: Linear discriminant analysis Effect size
MAFFT: Multiple alignment using fast fourier transform
OTU: Operational taxonomic units
PCoA: Principal coordinate analysis
PERMANOVA: Permutational multivariate analysis of variance
QIIME2: Quantitative insights into microbial ecology
rRNA: Ribosomal Ribonucleic acid
RStudio IDE: Integrated development environment
WUF: Weighted UniFrac metric
